# Basic Psychological Needs Satisfaction and Frustration, Stress, and Sports Injury Among University Athletes: A Four-Wave Prospective Survey

**DOI:** 10.3389/fpsyg.2019.00665

**Published:** 2019-03-26

**Authors:** Chunxiao Li, Andreas Ivarsson, Lawrence T. Lam, Jian Sun

**Affiliations:** ^1^Physical Education and Sports Science Academic Group, Nanyang Technological University, Singapore, Singapore; ^2^Department of Health and Physical Education, The Education University of Hong Kong, Tai Po, Hong Kong; ^3^School of Health and Welfare, Halmstad University, Halmstad, Sweden; ^4^Tung Wah College, King’s Park, Hong Kong; ^5^Faculty of Health, University of Technology Sydney, Ultimo, NSW, Australia; ^6^Faculty of Athletic Training, Guangzhou Sport University, Guangzhou, China

**Keywords:** athlete, injury, self-determination theory, longitudinal design, stress

## Abstract

The prevalence of sports injury among athletes is rather high, suggesting the need to better understand the causes of sports injury, including the risk factors, for preventive purposes. Grounded in basic psychological needs theory (BPNT) and the Model of Stress and Athletic Injury, the aim of this four-wave prospective survey study was to investigate the relationships among basic psychological needs satisfaction and frustration, stress responses, and sports injury. Study variables, including basic psychological need satisfaction/frustration, and perceived stress, were measured using a survey from 112 university athletes at the 1st, 2nd, and 3rd months of the study. Sports injury was assessed using a self-report form at the 2nd, 3rd, and 4th months of study. Results of the Bayesian multilevel analysis showed that basic psychological need satisfaction negatively predicted sports injuries, whereas stress was a positive predictor. In addition, basic psychological need satisfaction had an indirect effect on injury occurrence via stress. However, basic psychological need frustration did not predict sports injury. BPNT is a viable model to provide additional explanations to psychological risk factors of injury. Intervention programs may be formulated based on the evidence obtained on the model.

## Introduction

Sports injury is defined as any physical complaints sustained by an athlete as a result of training or competition, despite the need for medical attention or time loss from sports participation (Brink et al., 2010). It is common for athletes to sustain some sports injuries. For example, 51% of elite athletes reported at least one sports related injury over the last 12 months in China (Li et al., 2015). Also, injury rates were around 80% per year among Swedish elite soccer players (Hägglund et al., 2009). Sports injury will result in a lot of negative consequences such as pain, ill-being, poorer sport performance, and increased costs to health care system (Hägglund et al., 2013; Moesch et al., 2018). To this end, injury prevention has been a significant issue and risk factors of sports injury should be identified before injury prevention programs can be developed (Bahr, 2016).

Physiologically and biomechanically based research has dominated the area of sports-related injury research (Almeida et al., 2014). For example, a number of physiological and biomechanical related predictors of sports injury such as joint instability, muscle strength, range of motion, and postural stability have been identified (Bahr and Holme, 2003). Over the past two decades, an increasing number of studies has examined psychological predictors of sports injury and psychosocial factors such as competitive anxiety and emotional states were found to predict injury occurrence (Junge, 2000; Ivarsson et al., 2017; Singh and Conroy, 2017). Undoubtedly, it is of significance to conduct theoretically based research to examine the psychological risk factors of sports injury to interpret and apply the findings. Andersen and Williams’s Model of Stress and Athletic Injury (Williams and Andersen, 1998) is the most influential psychological model that has been developed to explain sports injury (Appaneal and Perna, 2014). Basic psychological needs theory (BPNT; Deci and Ryan, 2000) may be also a viable model to provide additional explanations to sports injury. The utility of this model in explaining mental and physical health has been well documented (see Ng et al., 2012). Guided by these two models, this prospective survey aimed to examine psychological risk factors of sports injury.

BPNT (Deci and Ryan, 2000) posits the universal existence of the three basic psychological needs in human being, which are autonomy (i.e., the need to experience volition and choice), competence (i.e., the need to feel competent and have capacities to accomplish goals), and relatedness (i.e., the need to experience interpersonal connection and caring; Deci and Ryan, 2000). BPNT also posits that the satisfaction of these basic psychological needs is universally essential for positive human growth and functioning. Autonomy, competence, and relatedness are specific and essential nutrients for thriving such as sport achievement and positive affect. However, a low level of satisfaction in these three basic psychological needs is expected to hamper growth. It could be even more harmful and pathogenic if these needs are frustrated (i.e., need frustration). To illustrate, athletes may feel low relatedness to coaches in their training setting (low relatedness). But athletes can also be actively excluded by their coaches (relatedness frustration). Thus, a low level of need satisfaction is different from need frustration (Bartholomew et al., 2011; Vansteenkiste and Ryan, 2013). According to BPNT (Deci and Ryan, 2000), need frustration will result in psychological maladjustment and even psychopathology such as burnout and illness (Li et al., 2013; Vansteenkiste and Ryan, 2013). To this end, it is possible that basic psychological needs satisfaction and frustration are potential risk factors of sports injury. However, there is little BPNT-based studies in the sports injury-related literature. This is an area worthy for further pursuit.

Different from the tenet of BPNT, Andersen and Williams’s Model of Stress and Athletic Injury (Williams and Andersen, 1998) posits that personality, history of stressors, and coping resources will impact on magnitude of stress responses of the athlete when he/she is exposed to a potentially stressful situation. The stress responses can be physiological, psychological, or both (e.g., muscle tension, distractibility, and perceptual narrowing). These responses could potentially increase the risk of sports injury (Williams and Andersen, 1998). Some recent systematic reviews provided evidence to support the notion that stress responses were positively related to injury rates (Ivarsson et al., 2017; Singh and Conroy, 2017). However, the Stress and Athletic Injury model has its drawback. Several potential risk factors of sports injury, such as motivation and emotional states, have been suggested to be missing from the model (Ivarsson et al., 2017), leaving rooms for the inclusion of other possible models as an explanatory framework for sports injury.

BPNT (Deci and Ryan, 2000) may be a viable model for providing additional explanations to sports injuries. For example, there is strong evidence showing that basic psychological needs satisfaction and frustration influence motivational and emotional outcomes (Bartholomew et al., 2011; Vansteenkiste and Ryan, 2013; Rodrigues et al., 2018; Teixeira et al., 2018), which are potential risk factors of sport injury. These are the missing components in the Andersen and Williams’s model (Hackfort and Kleinert, 2007). In addition, basic psychological need satisfaction has been considered as a coping resource (i.e., a predictor of sports injury as depicted in Andersen and Williams’s model). In line with the tenets of BPNT (Deci and Ryan, 2000), it is expected that athletes are likely to view and respond to the demands positively when their basic psychological needs are satisfied. For example, athletes who feel in control, competent, and supported by significant others (i.e., the level of basic psychological need satisfaction), will be capable to appraise and respond to stressful events positively.

On the other hand, when athletes’ needs are frustrated, they may appraise the demands as a threat to oneself and provide maladaptive responses (e.g., increased stress level). In a two-wave prospective study, it was showed that basic psychological need satisfaction measured at the baseline negatively predicted stress responses 1 month later among 61 full-time dancers (Quested et al., 2011). More studies with a true longitudinal design (i.e., at least three waves) are needed to replicate their finding to provide more rigorous evidence about the relationship between basic psychological need satisfaction/frustration and stress response as well as to understand the temporal process between risk factors and injury outcomes (Singer and Willett, 2003). Furthermore, the role of basic psychological need satisfaction/frustration in the relationship between stress and subsequent occurrence of sports injury is yet to be investigated. This is relevant to our understanding of the underlying process on how these variables are related to each other, which may contribute to theory building or refinement.

**FIGURE 1:**
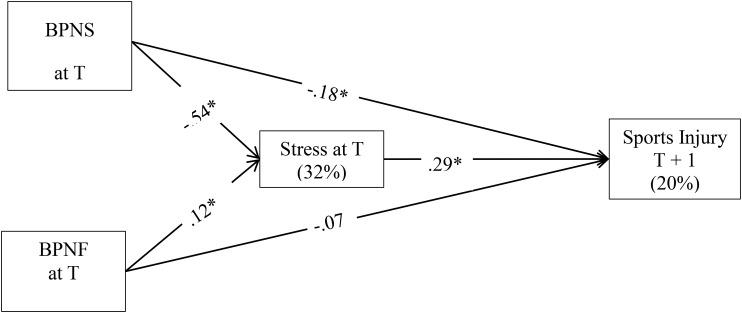
Hypothesized model with standardized path estimates. ^∗^Credible structural path; BPNS, basic psychological need satisfaction; BPNF, basic psychological need frustration; T, variable measured at Time T; T + 1, variable measured at the next occasion.

In summary, very little is known regarding the relationships among basic psychological need satisfaction/frustration, stress, and sports injuries as well as the utility of BPNT in this context. Applying BPNT and the Model of Stress and Athletic Injury in this context will advance our current knowledge on psychological risk factors of sports injuries and help practitioners (e.g., coaches and trainers) to design theory based injury prevention programs. This research therefore aims to investigate the relationships among basic psychological need satisfaction/frustration, stress, and sports injuries among university athletes through the lens of these two models. Furthermore, to capture how potential changes in level of stress and motivation might influence the risk of injury, a within-person approach will be used. By using such an approach it is possible to test the “when” question (e.g., what happens when an individual’s stress level increases; Zawadzki et al., 2015). According to BPNT (Deci and Ryan, 2000) and the Model of Stress and Athletic Injury (Williams and Andersen, 1998), it was hypothesized that basic psychological need satisfaction would negatively predict sports injuries while basic psychological need frustration would be a positive predictor (Hypotheses 1 and 2). In addition, it was hypothesized that stress would positively predict sports injuries (Hypothesis 3). Finally, we expected basic psychological need satisfaction/frustration to have an indirect effect on sports injuries via stress (Hypothesis 4; see Figure 1).

## Materials and Methods

### Participants

Athletes (*n* = 112; 61 males and 51 females) from a public university participated in this study. Participants had a mean age of 21.10 (*SD* = 1.99) years. They were recruited from seven sports teams, representing four team sports (i.e., basketball, handball, soccer, and rugby). On average, participants involved in their sport for 7.31 (*SD* = 3.95) years and trained 8.81 h (*SD* = 4.50) per week.

### Measures

The survey form included several demographic items (i.e., age, gender, sport, years of sports participation, and hours of training), two psychological predictors (i.e., basic psychological need satisfaction/frustration and perceived stress), and one major outcome (i.e., sports injuries).

#### Basic Psychological Needs Satisfaction and Frustration

The Chinese version of the Basic Psychological Needs Satisfaction and Frustration Scale developed and validated by Chen et al. (2015) was used to measure participants’ general basic psychological needs satisfaction and frustration over the past month. This scale consists of six 4-item subscales tapping into autonomy, relatedness, and competence satisfaction and frustration (e.g., “I feel excluded from the group I want to belong to”). Participants rated the scale items at months 1, 2, and 3 on a 5-point Likert scale (1, not true at all; 5, completely true). In this research, the basic psychological needs satisfaction/frustration subscales showed good Cronbach’s alpha reliability across the three measurement points (0.82 to 0.91). Overall basic psychological need satisfaction scores and overall basic psychological need frustration scores were computed for further analyses.

#### Perceived Stress

The Chinese version of the Perceived Stress Scale (Cohen et al., 1983) was used to assess the intensity of participants’ life stress during the last month. The validity and reliability of the scale have been examined with Chinese populations (e.g., Leung et al., 2010). The scale consists of 10 items (e.g., “In the last month, how often have you been upset because of something that happened unexpectedly?”). Participants rated the items on a five-point Likert scale (0, never; 4, very often) at months 1, 2, and 3. Cronbach’s alpha reliability was good across the three waves with the current sample (0.72, 0.75, and 0.74). A total scale score was calculated for further analyses.

#### Sports Injuries

An injury was counted if it resulted in an athlete to stop, limit, or modify sports participation for at least 1 day (Lysens et al., 1991). Injuries were evaluated by the medical staff and athletes and a recurrent injury was only counted once. Similar to early research (Bjørneboe et al., 2011), participants were invited to report sports-related injuries over the last month at months 2, 3, and 4 (“Did you experience a sports-related injury last month”; “Is that a new or recurrent injury”) using a 2-point rating scale (1, yes; 0, no).

### Procedure

The study was conducted in accordance with the Declaration of Helsinki. In addition, the study procedure of this research was approved by the Human Research Ethics Committee of the Education University of Hong Kong. Athletes from a public university in Hong Kong were invited to participate in this study. Upon obtaining participants’ written informed consent, the survey form was then distributed to them in quiet classrooms or sports halls under researchers’ supervision. Data were collected once per month in September (start of in-season), October, November, and December 2017 (toward the end of in-season). Participants’ basic psychological need satisfaction/frustration and stress were measured at months 1, 2, and 3 and their self-reported sport injuries were obtained at months 2, 3, and 4. For all the administration occasions, participants were encouraged to provide honest responses. Special emphasis was placed on confidentiality and no mandatory participation. To increase response rates, each participant received a cash coupon (US$19) after completing the whole survey. The response rates at months 1, 2, 3, and 4 were 100% (*n* = 112), 73.2% (*n* = 82), 77.7% (*n* = 87), and 89.3% (*n* = 100), respectively.

### Data Analysis

We performed all analyses within the Bayesian paradigm. Bayesian statistics are, in comparison to frequentist statistics, based on different assumptions (for more information about the differences between Bayesian and Frequentist statistics see, for example, van de Schoot and Depaoli, 2014; Stenling et al., 2015). Previous research has highlighted that Bayesian statistics have some advantages over Frequentist statistics. One of which that is relevant to the present study is no restrictive normality assumptions are imposed on sampling distributions of estimates and depend less on asymptotic theory. Because of these less restrictive assumptions, the odds of producing reliable results even with small samples are higher in comparison to the more stringent assumptions that the Frequentist statistics are based on (Song and Lee, 2012). Also, related to the less restrictive assumptions Bayesian estimation is recommended to perform mediation analyses (e.g., Yuan and MacKinnon, 2009).

Descriptive statistics were calculated using JASP software (0.8.5; Love et al., 2015). For the zero-order correlation analysis, a Bayes Factor (BF) was calculated for each of the relationships. The BF quantifies the evidence toward the alternative hypothesis in comparison to the null hypothesis. Based on previous recommendations (Etz and Vandekerckhove, 2016), a BF value above 10 indicates strong support for the alternative hypothesis (i.e., there is a statistical relationship between the two variables).

Three two-level path analyses, using the Bayesian estimator, were conducted to test the models related to Hypotheses 1–4. All the path analyses were conducted in *M*plus 8.0 (Muthén and Muthén, 1998/2018). In testing Hypotheses 1–3, the psychological data for each of the three variables (i.e., basic psychological need satisfaction/frustration and stress) from each time-point were used to prospectively predict sports injuries in the following month at the within-person level. To test Hypothesis 4, a within-subject mediation analysis was performed. In this analysis, we tested the indirect effect of basic psychological needs satisfaction and frustration on injury occurrence in the following month via stress (see Figure 1). The number of clusters (*n* = 112) was generally adequate for two-level path analysis (McNeish and Stapleton, 2016).

We used the Markov Chain Monte Carlo (MCMC) simulation procedures with a Gibbs sampler to generate credible parameter values for all the path analyses. We ran all the models using 100 000 iterations (50 000 burn-in by default), and we used every 10th iteration to reduce autocorrelation between MCMC draws. In line with previous recommendations, a potential scale reduction factor around 1 indicates substantial evidence of convergence (Kaplan and Depaoli, 2012). We evaluated model fit based on the generated posterior predictive *p* (PP*p*) value in combination with the 95% confidence intervals. A PP*p* value around 0.50 together with its 95% confidence intervals centering 0 are considered as an indication of good model fit (Muthén and Asparouhov, 2012).

A 95% credibility interval (CI) was estimated for each parameter specified in the analyses. The CI indicates the probability that, given the observed data, the value of the specified parameter lies between the upper and lower bound (Zyphur and Oswald, 2015). If the 95% CI around the parameter estimate did not include zero, we considered it to be a credible parameter estimate (i.e., we could reject the null hypothesis of no effect; cf. Zyphur and Oswald, 2015). Default priors in *M*plus were used in all the path analyses.

## Results

### Descriptive Statistics

On average, the participants reported high levels of basic psychological need satisfaction as well as moderate levels of basic psychological need frustration and perceived stress across the three measurement points. They also reported 126 sports injuries during the study period. For more information about the descriptive results, see Table 1.

**Table 1:** Descriptive statistics, internal reliability, and correlation estimates together with their Bayes Factors among the study variables.

	1	2	3	4	5	6	7	8	9	10	11	12	13	14
(1) T1 BPNS	—													
(2) T2 BPNS	0.54(69439)	—												
(3) T3 BPNS	0.32(10.39)	0.35(7.13)	—											
(4) T1 BPNF	-0.32(47.19)	-0.27(2.50)	-0.17(0.47)	—										
(5) T2 BPNF	-0.22(0.90)	-0.37(45.08)	-0.37(12.40)	0.65(1.66ˆ8)	—									
(6) T3 BPNF	-0.27(2.74)	-0.24(0.86)	-0.51(33665)	0.51(46265)	0.74(2.65ˆ9)	—								
(7) T1 PS	-0.41(2006)	-0.16(0.38)	-0.22(1.03)	0.51(1.24ˆ6)	0.38(58.21)	0.31(7.98)	—							
(8) T2 PS	-0.16(0.37)	-0.30(5.27)	-0.37(12.68)	0.29(3.44)	0.49(4630)	0.45(109.11)	0.45 (687)	—						
(9) T3 PS	-0.33(16.62)	-0.30(2.42)	-0.35(31.77)	0.34(20.26)	0.38(17.41)	0.52(81721)	0.47 (3323)	0.52 (1260)	—					
(10) T2 Injury	-0.03(0.15)	-0.16(0.36)	-0.18(0.40)	0.15(0.31)	0.26(1.90)	0.15(0.30)	0.42 (187)	0.27 (2.39)	0.36 (7.99)	—				
(11) T3 Injury	-0.03(0.14)	-0.34(6.44)	-0.15(0.34)	-0.06(0.16)	0.40(26.90)	0.09(0.19)	0.10 (0.20)	0.41 (32.0)	0.25 (1.75)	0.46 (133)	—			
(12) T4 Injury	-0.15(0.35)	-0.13(0.25)	-0.18(0.47)	-0.07(0.15)	0.15(0.32)	0.12(0.24)	-0.07 (0.16)	0.09 (0.19)	-0.08 (0.18)	0.09 (0.20)	0.36 (32.3)	—		
(13) Age	0.24(3.18)	0.14(0.30)	0.16 (0.39)	0.02(0.12)	0.02(0.14)	-0.01(0.14)	0.03 (0.12)	0.10 (0.20)	0.07 (0.17)	0.02 (0.14)	0.04 (0.15)	-0.06 (0.15)	—	
(14) Training	-0.03(0.13)	0.04(0.15)	-0.12(0.24)	0.11(0.23)	0.02(0.14)	0.18(0.50)	0.06 (0.14)	-0.03 (0.15)	-0.03 (0.14)	0.16 (0.37)	-0.03 (0.14)	0.03 (0.13)	0.17 (0.56)	—
*M* (*SD*)	3.74 (0.37)	3.67 (0.39)	3.60 (0.45)	2.56 (0.61)	2.63 (0.66)	2.71 (0.55)	19.54 (4.80)	20.46 (4.18)	21.23 (4.36)	0.53 (0.50)	0.49 (0.50)	0.41 (0.50)	21.10 (1.99)	8.81 (4.50)
Potential range	1–5	1–5	1–5	1–5	1–5	1–5	0–40	0–40	0–40	0–1	0–1	0–1	NA	NA
Actual range	3–5	3–4	2–4	1–5	2–4	1–4	6–29	11–29	10–31	0–1	0–1	0–1	17–26	1.5–32
α	0.82	0.84	0.86	0.89	0.91	0.87	0.72	0.75	0.74	NA	NA	NA	NA	NA


*For each of the estimated relationships, the correlation estimate is presented together with the Bayes Factor, which is included in the parenthesis. A Bayes Factor above 10 is considered to indicate strong evidence for the alternative hypothesis (i.e., there is a statistical relationship between the two variables). BPNS, basic psychological need satisfaction; BPNF, basic psychological need frustration; PS, perceived stress; Training, average number of training hours per week; NA, not available*.

### Hypotheses 1–2

The model using basic psychological needs satisfaction and frustration from each time-point to prospectively predict sports injury showed good fit to the data (PP*p* = 0.50, 95% Confidence Interval = [-9.77, 10.11]). Sports injuries had a credible variance at the between-person level (λ = 1.11, 95% CI = [0.33, 2.94]). At the within-person level, the two independent variables could explain 9% of the variance in sport injury. More specifically, in line with Hypothesis 1, basic psychological need satisfaction was a negative credible predictor of sports injury the following month (β = -0.27, 95% CI = [-0.47, -0.05]). However, contrary to Hypothesis 2, basic psychological need frustration had no credible effect on sports injury the following month (β = -0.02, 95% CI = [-0.27, 0.22]).

### Hypothesis 3

The model using stress from each time-point to prospectively predict sports injury showed good fit to the data (PP*p* = 0.50, 95% Confidence Interval = [-9.09, 8.97]). Sports injury had a credible variance at between-person level (λ = 0.90, 95% CI = [0.29, 2.25]). At the within-person level, perceived stress was a positive credible predictor of sports injury the following month (β = 0.26, 95% CI = [0.04, 0.46]) and could explain 7.0% of the variance in sports injury. Therefore, Hypotheses 3 was supported.

### Hypothesis 4^1^

The model depicted in Figure 1 showed good fit to the data (PP*p* = 0.53, 95% Confidence Interval [-13.42, 12.92]). At the within-person level, basic psychological need satisfaction had an indirect effect on sports injury the following month via stress (*ab* = -0.41, 95% CI = [-0.83, -0.04]). More specifically, basic psychological need satisfaction had a negative credible effect on perceived stress (β = -0.54, 95% CI = [-0.61, -0.45]), which in turn, had a positive credible effect on sports injury (β = 0.29, 95% CI = [0.03, 0.52]). In addition, basic psychological need satisfaction had a negative credible effect on sports injury (β = -0.18, 95% CI = [-0.35, -0.01]). The two predictors together explained 11.4% of the variance in sports injury.

There was no credible indirect effect of basic psychological need frustration on sport injury in the following month via stress (*ab* = 0.06, 95% CI = [-0.001, 0.17]). The three independent variables could together, on within-person level, explain 20% of the variance in injuries the next month. Also, 32% of the variance in stress could be explained by basic psychological needs satisfaction and frustration. These findings were partially in line with Hypothesis 4.

## Discussion

Guided by BPNT and the Model of Stress and Athletic Injury, this four-wave prospective survey aimed to identify psychological predictors of sports injury. Specifically, we examined the relationships among basic psychological needs satisfaction/frustration, perceived stress, and sports injuries among university athletes. One significant contribution of this research is that sports injury is, for the first time, investigated as an outcome of basic psychological need satisfaction/frustration via the lens of BPNT (Deci and Ryan, 2000). We found that basic psychological need satisfaction/frustration was a significant predictor of stress. This result is consist with a number of previous studies, in which basic needs satisfaction and frustration were found to predict a wide range of outcomes such as life satisfaction, physical health, stress, and vitality (Bartholomew et al., 2011; Ng et al., 2012; Rodrigues et al., 2018; Teixeira et al., 2018). In line with the tenet of BPNT that the fulfillment of basic psychological needs is essential for positive human functioning (Deci and Ryan, 2000), basic psychological need satisfaction was found as a negative credible predictor of sports injury in the present study. However, basic psychological need frustration had no credible effect on sports injury in the following month. Our findings suggest that the manifestation of sports injury may be more related to the presence of basic psychological need satisfaction than the presence of basic psychological need frustration. Given this is the first study to investigate the relationship between needs frustration and sports injury, further evidence is needed to confirm this result.

In addition to basic psychological need satisfaction, stress was identified as another risk factor of sports injury in our study. This result is parallel to the finding of a recent meta-analysis (Ivarsson et al., 2017) and the Model of Stress and Athletic Injury (Williams and Andersen, 1998). According to Williams and Andersen (1998), stress will not directly result in sports injury, rather physiological or psychological responses (e.g., reduced attention, decreased neuromuscular control, and negative immune responses) induced by elevated stress will directly cause sports injury. Williams and Andersen’s (1998) model also posits that there are three major antecedents of stress, including personality, history of stressors, and coping resources. Basic psychological need satisfaction can be classified as a coping resource (Deci and Ryan, 2000), which was found to negatively predict stress in the current research. This result suggests that athletes with different levels of basic psychological need satisfaction will react differently to stressors. In consistent with previous research (Quested et al., 2011), athletes who have a high level of basic psychological need satisfaction, relative to low level ones, are more likely to react to stressful events in a positive way (e.g., more stable emotions and lower muscle aches). Basic psychological need satisfaction represents a critical resource for athletes to process stressful events openly and choicefully, cope with challenges confidently, and relate to significant others to get through adversity (Vansteenkiste and Ryan, 2013). Thus, basic psychological need satisfaction had a negative association with stress. In a similar vein, basic psychological need frustration had a positive association with stress.

The third finding of this research is that stress partially accounted for the relationship between basic psychological need satisfaction and sports injury. In other words, direct path from basic psychological need satisfaction to sports injury was still significant after accounting for the role of stress. This finding provides preliminary evidence about the relationships among these three studied variables and is of importance for theory building. This finding suggests that basic psychological needs can be viewed as a coping resource that might reduce the risk of injury due to a decrease in the magnitude of stress responses (Williams and Andersen, 1998). The finding also suggests the unique contribution of basic psychological need satisfaction in explaining sports injury. This could possibly be due to the fact that basic psychological need satisfaction is closely linked to motivational and emotional outcomes that are believed to predict sports injury (Hackfort and Kleinert, 2007). Thus, BPNT is a viable model for providing supplementary explanations to the Model of Stress and Athletic Injury. All the psychological predictors together explained 20.0% of the variance in sports injuries, which is interpreted as a moderate to substantial effect (Cohen, 1992). Based on these positive findings, sports injury prevention programs may integrate training components (e.g., control of emotions, imagery, mindfulness, and self-talk) to reduce athletes’ stress and fulfill their basic psychological needs. For example, using imagery and self-talk skills has been found to decrease stress and increase self-confidence (Hatzigeorgiadis et al., 2009). Integrating mindfulness-based training (e.g., body scan and sitting meditation) can be also useful given its effectiveness in both stress reduction and fulfillment of basic psychological needs among athletes (Vansteenkiste and Ryan, 2013; Petterson and Olson, 2017; Li et al., 2019).

Despite the significant findings and practical implications, there are some limitations in this study. First, the prospective survey design used does not allow for causal inferences among the studied variables. For example, stress may not only result in, but may be also caused by sports injury. Second, a fairly homogenous sample (i.e., university team sport athletes) was recruited, future research should examine whether the present findings can be generalized into other samples such as school athletes or individual sports players. Finally, exclusive reliance on self-report measures is likely to inflate observed correlations because of the shared method variance. As such, objective measures (e.g., cortisol also known as stress hormone) may be applied in future investigations to overcome this limitation.

## Conclusion

In conclusion, this multi-wave prospective survey underscores the relationships among basic psychological need satisfaction/frustration, stress, and occurrence of sports injury among university athletes. Our findings indicate that athletes are likely to experience a sports injury if they have a low level of basic psychological need satisfaction and a high level of stress. Perceived stress partially accounts for the relationship between basic psychological need satisfaction and sports injury, indicating that stress may explain the underlying process between needs satisfaction to sports injury, and BPNT is a viable model to provide additional explanations to the Model of Stress and Athletic Injury. These findings suggest that an intervention program designed for both basic psychological need satisfaction enhancement and stress reduction may be effective in the prevention of sports injury among university students.

## Data Availability

All datasets generated for this study are included in the manuscript and/or the supplementary files.

## Author Contributions

CL, AI, and LL conceived the overall study design. AI and CL analyzed the data. All the authors contributed to the manuscript writing.

## Conflict of Interest Statement

The authors declare that the research was conducted in the absence of any commercial or financial relationships that could be construed as a potential conflict of interest.

## Footnotes

1 As an exploratory analysis, gender was included in the models testing Hypothesis 4 as a moderator, and that did not improve the model fit.

## References

[B1] Almeida P. H. G. L. D. Olmedilla A. Rubio V. J. Palou P. (2014). Psychology in the realm of sport injury: what it is all about. *Revista de Psicología del Deporte* 23 395–400.

[B2] Appaneal R. N. Perna F. M. (2014). “Biopsychosocial model of injury,” in *Encyclopedia of Sport and Exercise Psychology* eds Eklund R. Tenenbaum G. (Thousand Oaks, CA: Sage Publications) 74–77.

[B3] Bahr R. (2016). Why screening tests to predict injury do not work and probably never will: a critical review. *Br. J. Sports Med.* 50 776–780. 10.1136/bjsports-2016-096256 27095747

[B4] Bahr R. Holme I. (2003). Risk factors for sports injuries. Methodological approach. *Br. J. Sports Med.* 37 384–392. 10.1136/bjsm.37.5.384 14514527 PMC1751357

[B5] Bartholomew K. J. Ntoumanis N. Ryan R. M. Thøgersen-Ntoumani C. (2011). Psychological need thwarting in the sport context: assessing the darker side of athletic experience. *J. Sport Exerc. Psych.* 33 75–102. 10.1123/jsep.33.1.75 21451172

[B6] Bjørneboe J. Flørenes T. W. Bahr R. Andersen T. E. (2011). Injury surveillance in male professional football: is medical staff reporting complete and accurate? *Scand. J. Med. Sci. Sports* 21 713–720. 10.1111/j.1600-0838.2009.01085.x 20459470

[B7] Brink M. S. Visscher C. Arends S. Zwerver J. Post W. J. Lemmink K. A. (2010). Monitoring stress and recovery: new insights for the prevention of injuries and illnesses in elite youth soccer players. *Br. J. Sports. Med.* 44 809–815. 10.1136/bjsm.2009.069476 20511621

[B8] Chen B. Vansteenkiste M. Beyers W. Boone L. Deci E. L. Van der Kaap-Deeder J. (2015). Basic psychological need satisfaction, need frustration, and need strength across four cultures. *Motiv. Emot.* 39 216–236. 10.1007/s11031-014-9450-1

[B9] Cohen J. (1992). A power primer. *Psychol. Bull.* 112 155–159. 10.1037/0033-2909.112.1.155 19565683

[B10] Cohen S. Kamarck T. Mermelstein R. (1983). A global measure of perceived stress. *J. Health Soc. Behav.* 24 385–396. 10.1037/0033-2909.112.1.155 6668417

[B11] Deci E. L. Ryan R. M. (2000). The” what” and” why” of goal pursuits: human needs the self-determination of behavior. *Psychol. Inq.* 11 227–268. 10.1207/S15327965PLI1104_01 27055568

[B12] Etz A. Vandekerckhove J. (2016). A bayesian perspective on the reproducibility project: psychology. *PLoS One* 11:e0149794. 10.1371/journal.pone.0149794 26919473 PMC4769355

[B13] Hackfort D. Kleinert J. (2007). Research on sport injury development: former and future approaches from action theory perspective. *Int. J. Sport Exerc. Psychol.* 5 324–339. 10.1080/1612197X.2007.9671839

[B14] Hägglund M. Waldén M. Ekstrand J. (2009). Injuries among male and female elite football players. *Scand. J. Med. Sci. Sports* 19 819–827. 10.1111/j.1600-0838.2008.00861.x 18980604

[B15] Hägglund M. Waldén M. Magnusson H. Kristenson K. Bengtsson H. Ekstrand J. (2013). Injuries affect team performance negatively in professional football: an 11-year follow-up of the UEFA Champions League injury study. *Br. J. Sports Med.* 47 738–742. 10.1136/bjsports-2013-092215 23645832

[B16] Hatzigeorgiadis A. Zourbanos N. Mpoumpaki S. Theodorakis Y. (2009). Mechanisms underlying the self-talk–performance relationship: the effects of motivational self-talk on self-confidence and anxiety. *Psychol. Sport Exerc.* 10 186–192. 10.1016/j.psychsport.2008.07.009

[B17] Ivarsson A. Johnson U. Andersen M. B. Tranaeus U. Stenling A. Lindwall M. (2017). Psychosocial factors and sport injuries: meta-analyses for prediction and prevention. *Sports Med.* 47 353–365. 10.1007/s40279-016-0578-x 27406221

[B18] Junge A. (2000). The influence of psychological factors on sports injuries. *Am. J. Sports Med.* 28 S10–S15. 10.1177/28.suppl_5.s-10 11032102

[B19] Kaplan D. Depaoli S. (2012). “Bayesian structural equation modeling,” in *Handbook of Structural Equation Modeling* ed. Hoyle R. H. (New York, NY: Guilford Press) 650–673. 10.1177/28.suppl_5.s-10

[B20] Leung D. Y. Lam T. H. Chan S. S. (2010). Three versions of perceived stress scale: validation in a sample of chinese cardiac patients who smoke. *BMC Public Health* 10:513. 10.1186/1471-2458-10-513 20735860 PMC2939644

[B21] Li C. Lam T. T. Wu Y. (2015). Sports related injuries among Chinese Paralympic athletes. *Eur. J. Adapt. Phys. Activ.* 8 37–43. 10.5507/euj.2015.007

[B22] Li C. Wang C. K. J. Pyun D. Y. Kee Y. H. (2013). Burnout and its relations with basic psychological needs motivation among athletes: a systematic review and meta-analysis. *Psychol. Sport Exerc.* 14 692–700. 10.1016/j.psychsport.2013.04.009

[B23] Li C. Zhu Y. Zhang M. Gustafsson H. Chen T. (2019). Mindfulness and athlete burnout: a systematic review and meta-analysis. *Int. J. Environ. Res. Public Health* 16:449. 10.3390/ijerph16030449 30717450 PMC6388258

[B24] Love J. Selker R. Marsman M. Jamil T. Dropmann D. Verhagen A. J. (2015). *JASP (Version 0.8.5)*.

[B25] Lysens R. De Weerdt W. Nieuwboer A. (1991). Factors associated with injury proneness. *Sports Med.* 12 281–289. 10.2165/00007256-199112050-00001 1763246

[B26] McNeish D. M. Stapleton L. M. (2016). The effects of small sample size on two-level model estimates: a review and illustration. *Educ. Psychol. Rev.* 28 295–314. 10.1007/s10648-014-9287-x

[B27] Moesch K. Kenttä G. Kleinert J. Quignon-Fleuret C. Cecil S. Bertollo M. (2018). FEPSAC position statement: mental health disorders in elite athletes and models of service provision. *Psychol. Sport Exerc.* 38 61–71. 10.1016/j.psychsport.2018.05.013

[B28] Muthén B. Asparouhov T. (2012). Bayesian structural equation modeling: a more flexible representation of substantive theory. *Psychol. Methods* 17 313–335. 10.1037/a0026802 22962886

[B29] Muthén L. K. Muthén B. O. (1998/2018). *Mplus User’s Guide* 8th Edn. Los Angeles, CA: Muthén & Muthén.

[B30] Ng J. Y. Ntoumanis N. Thøgersen-Ntoumani C. Deci E. L. Ryan R. M. Duda J. L. (2012). Self-determination theory applied to health contexts: a meta- analysis. *Perspect. Psychol. Sci.* 7 325–340. 10.1177/1745691612447309 26168470

[B31] Petterson H. Olson B. L. (2017). Effects of mindfulness-based interventions in high school and college athletes for reducing stress and injury, and improving quality of life. *J. Sport Rehabil.* 26 578–587. 10.1123/jsr.2016-0047 27632857

[B32] Quested E. Bosch J. A. Burns V. E. Cumming J. Ntoumanis N. Duda J. L. (2011). Basic psychological need satisfaction, stress-related appraisals, and dancers’ cortisol and anxiety responses. *J. Sport Exerc. Psychol.* 33 828–846. 10.1123/jsep.33.6.828 22262707

[B33] Rodrigues F. Bento T. Cid L. Neiva H. Teixeira D. S. Moutão J. (2018). Can interpersonal behavior influence the persistence and adherence to physical exercise practice in adults? Systematic review. *Front. Psychol.* 9:2141. 10.3389/fpsyg.2018.02141 30459690 PMC6232376

[B34] Singer J. D. Willett J. B. (2003). *Applied Longitudinal Data Analysis: Modeling Change and Event Occurrence*. New York, NY: Oxford University Press 10.1093/acprof:oso/9780195152968.001.0001

[B35] Singh H. Conroy D. E. (2017). Systematic review of stress-related injury vulnerability in athletic and occupational contexts. *Psychol. Sport Exerc.* 33 37–44. 10.1016/j.psychsport.2017.08.001

[B36] Song X. Y. Lee S. Y. (2012). *Basic and Advanced Bayesian Structural Equation Modeling: With Applications in the Medical and Behavioral Sciences*. Hoboken, NJ: Wiley 10.1002/9781118358887

[B37] Stenling A. Ivarsson A. Johnson U. Lindwall M. (2015). Bayesian structural equation modeling in sport and exercise psychology. *J. Sport Exerc. Psychol.* 37 410–420. 10.1123/jsep.2014-0330 26442771

[B38] Teixeira D. S. SIlva M. N. Palmeira A. L. (2018). How does frustration make you feel? A motivational analysis in exercise context. *Motiv. Emot.* 42 419–428. 10.1007/s11031-018-9690-6

[B39] van de Schoot R. Depaoli S. (2014). Bayesian analyses: where to start and what to report. *Eur. Health Psychol.* 16 75–84.

[B40] Vansteenkiste M. Ryan R. M. (2013). On psychological growth and vulnerability: basic psychological need satisfaction and need frustration as a unifying principle. *J. Psychother. Integr.* 23 263–280. 10.1037/a0032359

[B41] Williams J. M. Andersen M. B. (1998). Psychosocial antecedents of sport injury: review and critique of the stress and injury model. *J. Appl. Sport Psychol.* 10 5–25. 10.1080/10413209808406375

[B42] Yuan Y. MacKinnon D. P. (2009). Bayesian mediation analysis. *Psychol. Methods* 14 301–322. 10.1037/a0016972 19968395 PMC2885293

[B43] Zawadzki M. J. Smyth J. M. Costigan H. J. (2015). Real-time associations between engaging in leisure and daily health and well-being. *Ann. Behav. Med.* 49 605–615. 10.1007/s12160-015-9694-3 25724635

[B44] Zyphur M. J. Oswald F. L. (2015). Bayesian estimation and inference: a user’s guide. *J. Manage.* 41 390–420. 10.1177/0149206313501200

